# Commencing colorectal cancer screening at age 45 years in U.S. racial groups

**DOI:** 10.3389/fonc.2022.966998

**Published:** 2022-07-22

**Authors:** John M. Carethers

**Affiliations:** Division of Gastroenterology and Hepatology, Department of Internal Medicine, and Department of Human Genetics and Rogel Cancer Center, University of Michigan, Ann Arbor, MI, United States

**Keywords:** colon cancer screening, cancer disparity, early onset colon adenocarcinoma, African American (AA), screening age, colonoscopy, fecal immunochemical (FIT) test

## Abstract

Screening for colorectal cancer (CRC) is cost-effective for reducing its mortality among the average-risk population. In the US, CRC incidence and mortality differ among racial/ethnic groups, with non-Hispanic Blacks (NHB) and American Indian/Alaska Natives showing highest incidence and mortality and earlier presentation. Since 2005, some professional societies have recommended CRC screening for NHB to commence at 45 years or earlier; this was not implemented due to lack of recommendation from key groups that influence insurance payment coverage. In 2017 the highly influential U.S. Multi-Society Task Force for Colorectal Cancer recommended screening to commence at 45 years for NHB; this recommendation was supplanted by data showing an increase in early-onset CRCs in non-Hispanic Whites approaching the under-50-year rates observed for NHB. Subsequently the American Cancer Society and the USPSTF recommended that the entire average-risk population move to commence CRC screening at 45 years. Implementing screening in 45–49-year-olds has its challenges as younger groups compared with older groups participate less in preventive care. The US had made extensive progress pre-COVID-19 in closing the disparity gap for CRC screening in NHB above age 50 years; implementing screening at younger ages will take ingenuity, foresight, and creative strategy to reach a broader-aged population while preventing widening the screening disparity gap. Approaches such as navigation for non-invasive and minimally invasive CRC screening tests, removal of financial barriers such as co-pays, and complete follow up to abnormal non-invasive screening tests will need to become the norm for broad implementation and success across all racial/ethnic groups.

## Introduction

Implementation of colorectal cancer (CRC) screening by non-invasive or minimally invasive means is associated with reduced mortality from CRC. Screening identifies average-risk persons who might harbor neoplasia and identifies patients with CRCs at potentially curable stages (1, 2). CRCs generally develop from precursor adenomas driven by well-described genetic alterations that may take 1 to 2 decades to manifest as cancer (3), affording time to interrupt this process *via* polypectomy (4, 5). Completion of CRC screening after any abnormal test involves the use of colonoscopy. Although colonoscopy is the gold standard and enables polypectomy, there remain challenges for high-quality exams due to the need to have the patient travel to the medical exam with an accompanying person (because sedation is used), and desire for good bowel cleansing preparation. Detection of lesions at colonoscopy and prevention of death from CRC depends on the endoscopist’s adenoma detection rate (6). Colonoscopy can still miss right-sided lesions even though it is the best test to detect them, due to discernability of proximal lesions from normal mucosa and differing biology of right-sided lesions (4, 7, 8). Utilization of CRC screening assumes that persons at average-risk are recommended for the test by providers, the person completes the test, and both provider and patient follow-through on results of the test. The intention in the US is universal CRC screening of at-risk men and women; yet pre-COVID-19 screening utilization rates were 65% of the eligible US population, meaning one-third of eligible persons were not getting screened, elevating their risk (1).

The age to commence CRC screening was determined to be 50 years based on the epidemiology of CRC in the 1990s and results of randomized controlled trials of FOBT showing reduction in CRC incidence and identifying earlier-staged lesions (9, 10). In the general population, 95% of CRCs occurred after 50 years, with 5% occurring earlier (10). Guidelines emerged incorporating data from studies into consensus recommendations for those ≥50 years, including tests to use (**Table 1**), the importance of follow-up of abnormal tests, and differentiating average-risk from high-risk individuals (those with family history of cancer or heritable syndrome, inflammatory bowel disease, or prior identified neoplasia) (11). Race as a risk factor (*see below*) was not considered in any major guidelines until 2017 (11) despite evidence for higher risk for CRC in specific groups. Recent data identifies a shift in age distribution of CRC for the general population, with 88% of CRCs occurring after 50 years and 12% occurring under 50 years, a more than doubling of early-onset cancers over the past 30 years (12–16). This shift is observed in persons born after 1960 with the largest group under 50 years showing increase being 45-49-year-olds (12, 13). This increase in early-onset CRC is environmental and not genetic, with several metabolic factors as possible etiologies (13, 16). Due to increased proportion of persons with CRC under 50 years, professional organizations began to modify recommendations for CRC screening commencement to 45 years (17, 18). The key recommendation for commencing screening at age 45 years came from the USPSTF in 2021, the group that CMS and other insurers generally follow due to their rigorous analytic methods and modeling.

**Table 1:** Currently available, FDA-approved tests for colorectal cancer screening (11).

Rank Order of Preference	Screening Test	Frequency if no findings
Tier 1	Colonoscopy	Every 10 years
	Fecal Immunochemical Test (FIT)	Annual
Tier 2	Fecal DNA Test combined with FIT	Every 3 years
	CT Colonography	Every 5 years
	Flexible sigmoidoscopy	Every 5 years (10 years with FIT)
Tier 3	Capsule colonoscopy	Every 5 years
Relatively obsolete	Guaiac-based Fecal Occult Blood Test (FOBT)	Replaced by FIT
	Barium Enema	Replaced by CT Colonography
Not recommended	Methylated *SEPTIN9* blood test	–

Fecal DNA Test is also known as multitarget stool DNA test (mt-sDNA) or FIT-DNA test.

## Epidemiology of CRC in racial groups

Initiation of CRC screening in the US was for the entire at-risk population, with age and family history as primary determinants for screening commencement (2, 9, 11). However, the US population is made up of diverse racial and ethnic groups, each showing varying CRC incidence and mortality. Until recently, the non-Hispanic Black (NHB) population has had the highest incidence and mortality from CRC among non-Hispanic Whites (NHW), Asian/Pacific Islanders, American Indian/Alaska Native (AI/AN), and Hispanics (2, 10, 19, 20), and has been consistently documented since before the 1990s (21–23). Implementation of CRC screening has lowered incidence and mortality rates for all races and ethnicities; however, disparity still exists for NHB (1, 2, 10, 20, 21). There are several factors that contribute to the disparity. First is underlying socioeconomic inequalities that dictates which zip code one lives, influencing accessibility to fresh produce and high availability of tobacco and alcohol, accessibility to preventive care, and predicts education attainment and level of employment. This, in turn, influences individual metabolic derangements over the lifetime, with alterations in gut microbiome and increased metabolic-induced inflammation. This, in turn, alters colonic cell proliferation and increases genetic mishaps, and likelihood for adenoma formation that can transform into CRC (20). This notion is solidified by observations that NHB have higher incidence of high-risk precursor adenomas (24, 25), and present 0-8 years younger with CRC than NHW (2, 10, 20). Since the 1990s, the proportion of CRCs under 50 years in NHB was 10.6%, about double the rate for NHW for that time period (2, 10, 20). Second, NHB show 7-15% higher prevalence of proximal CRCs (between cecum and splenic flexure) compared to NHW (2, 26), where sensitivity of the highest sensitive screening test, colonoscopy, is less than distal sites at detecting lesions (4). This finding parallels increased prevalence of proximal high-risk adenomas in NHB (24, 25). Earlier age of onset for high-risk adenomas and CRC in NHB coupled with increased prevalence of proximal neoplasia even with colonoscopy as the screening modality could amplify the disparity. Proximal CRCs in NHB are mostly microsatellite stable, with less prevalence of microsatellite unstable (MSI) CRCs compared to NHW (26). Consistent with this, there is no evidence that sessile serrated adenomas (SSAs), which are proximal and demonstrate MSI, are increased in NHB (2, 27, 28). The lower prevalence of MSI CRCs among NHB may itself contribute towards poor outcomes as MSI is associated with longer survival and eligibility for immune checkpoint inhibition therapy (29). Third, colonic microenvironment may be altered in NHB to favor CRC, with increased inflammation to generate inflammation-associated microsatellite alterations (30), which are associated with metastasis and poor survival (31–34). Indeed, use of NSAIDs had no deleterious consequences in older NHB CRC patients as compared to shorter survival for older NHW CRC patients (35). The cytotoxic immune cell response to cancer may be hampered in NHB CRCs compared to NHW (26, 36, 37). The colonic microbiome in NHB often show increase in sulfidogenic bacteria and pro-inflammatory *Fusobacterium* and *Enterobacter* species, all associated with neoplasia (38, 39). Lastly, there has been longstanding screening utilization disparity between racial/ethnic groups in the US, with NHB utilization 15% lower than NHW in the 2000s and 3% lower in 2018 (40). Navigated colonoscopic screening can eliminate incidence and mortality disparity (41). COVID-19 has showcased disparities in outcome, and shares similarities with disparities observed for CRC (42, 43). One consequence of COVID-19 was reduction in population CRC screening that is predicted to cause unnecessary cancer deaths over the next decade (44). Recovery of population-based CRC screening levels is likely to be uneven among racial/ethnic groups, with NHB recovering slower, widening the screening gap (44). Overall, there may be multiple components that contribute to CRC disparities in NHB.

In 2022, AI/AN demonstrate the highest CRC incidence among racial/ethnic groups in the US, overtaking the high rates observed for NHB (1) (**Table 2**). This observation is buttressed by a now-recognized propensity for AI/AN developing high-risk-adenomas (23). It may be only a few years that CRC mortality for AI/AN surpasses the high rates of NHB (1, 20). This observed incidence increase may be the result of longer life span from improved co-morbidities. CRC screening utilization for AI/AN was 59% in 2018, lower than that of NHB (40). These observations seem to replicate the disparity-driven conditions observed for NHB.

**Table 2:** Age-adjusted colorectal cancer incidence rates, 2014-2018, and age-adjusted colorectal cancer mortality, 2015-2019, among U.S. racial groups. Data are per 100,000 (adjusted to the 2000 US Census) (1).

	All	NH White	NH Black	Asian/PI	Am Indian/Alaska Native	Hispanic
**Incidence** Overall	36.5	36.1	42.6	29.0	49.2	32.8
Male	42.1	41.5	50.4	34.4	55.8	39.2
Female	31.6	31.3	37.1	24.6	43.9	27.6
*Early-onset (20-44 years)		6.7	7.9	6.3		
**Deaths** Overall	13.4	13.4	18.1	9.3	17.4	10.8
Male	16.0	15.8	22.7	11.1	21.3	13.7
Female	11.3	11.3	14.8	7.9	14.4	8.5

Data for early-onset colorectal cancer are age-adjusted colorectal cancer incidence rates, 2000-2012 (2000 US Standard Population) (15).

## Policy recommendations for commencing CRC screening in racial groups

Risk for CRC derives from factors that are potentially modifiable (diet, weight, physical activity, aspirin use, socioeconomic status, and screening utilization), and factors that are not modifiable (age, family history, race). Major components that determine commencement of CRC screening are age and family history, with subsequent screening intervals determined by findings on the initial screen and family history (9, 20). Race had not been included in CRC screening recommendations (until 2017) despite several decades of demonstrated disparity for incidence and mortality from CRC for NHB in particular (2, 20, 45–47).

**Figure 1:**
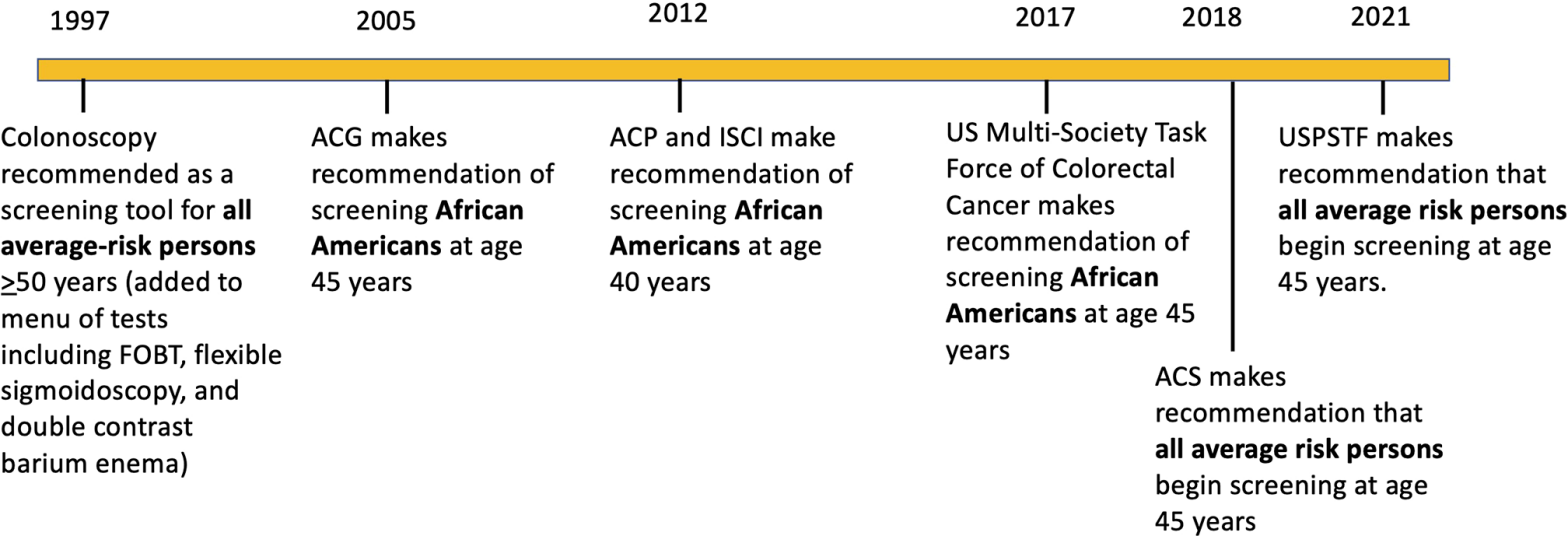
Timeline of colorectal screening recommendations for average risk persons and African Americans over the past 3 decades by various organizations (2, 9). ACG: American College of Gastroenterology; ACP, American College of Physicians; ICSI, Institute for Clinical Systems Improvement; ACS, American Cancer Society; USPSTF United States Preventative Services Task Force.

Because of the epidemiology observed for NHB for CRC, several organizations have made the recommendation to commence CRC screening in NHB at 45 years or earlier (2, 11, 46, 48) (**Figure 1**). The rationale for recommendation was shorter time between screening initiation and cancer formation in NHB (2). While the evidence was not deemed the highest-grade, the overall approach to CRC screening recommendations has generally included consideration of natural history and epidemiology for groups of individuals at risk. For instance, as with age, family history is a determinant when CRC screening commences (age 40 years) (11). Specific conditions are addressed in guidelines based on high-risk conditions, including presence of inflammatory bowel disease, Lynch syndrome, familial adenomatous polyposis, or personal history of colonic neoplasia (11). Generally, if a condition has propensity for earlier age of onset for CRC, shows higher morbidity and mortality at younger ages, and possesses higher prevalence of high-risk precursor adenomas (all of which describe the epidemiology of NHB patients with CRC), these observations should provide awareness and bestow rationale for adjusting the CRC screening initiation age (2). Earlier age commencement for CRC screening in NHB was recommended by ACG, ICSI, and ACP over the past 2 decades (11, 46, 48) (**Figure 1**). These organizations, while influential to large constituencies, do not compel insurers to provide payment coverage for earlier screening. In 2017 the highly influential MSTF made their first-ever recommendation to include race in their guidelines, recommending that NHB commence screening at 45 years (11). This recommendation greatly increased awareness, with some healthcare organizations such as Kaiser Permanente enacting screening for NHB at 45 years (49). Despite awareness, it did not immediately modify private and public insurer payment coverage.

The one modifiable item that providers (and patients) greatly influence is screening utilization. Modifying diet and physical activity are possible but are hard for most individuals to comply over a lifetime. Socioeconomic circumstances for individuals are not readily adjustable to modify CRC risk. Screening, however, is a great equalizer as it can reduce CRC risk *and* erase disparities for incidence and mortality if highly utilized. This was illustrated by the Delaware Cancer Consortium with navigated screening colonoscopies in 10,000 NHB and NHW patients (41). Comparing patients at the start with those at completion of the study, screening for average-risk NHB increased from 47.8% to 73.5% and for average-risk NHW individuals increased from 58.0% to 74.7%, benefiting both groups (41). For NHB, screening implementation reduced advanced CRCs from 78% to 40% and increased local stage CRCs from 15% to 50% (41). Importantly, CRC incidence dropped for both NHB and NHW, completely erasing incidence disparity. CRC mortality disparity was nearly erased, with NHB dropping from 31.27 to 18.35 deaths/100,000, and NHW dropping from 19.45 to 16.94 deaths/100,000 (41). Non-invasive screening can also eliminate disparities. Kaiser Permanente demonstrated increased screening utilization among NHB from 42% to 80% and among NHW from 40% to 83% from 2000 to 2015-2019 (50). CRC incidence dropped for NHW (135 to 78 cases/100,000 from 2009 to 2017-2019) and NHB (166 to 82 cases/100,000 from 2010 to 2017-2019), nearly eliminating incidence disparity (50). Likewise, CRC mortality disparity disappeared with reductions for NHW (33 to 20 cases/100,000 from 2007-2009 to 2017-2019) and NHB (54 to 21 cases/100,000 from 2007-2009 to 2017-2019) (50). These data show importance of CRC screening overall for all at-risk populations, as well as impact on health equity. The main factor for success and eliminating disparities in both studies is high utilization of screening across populations.

Initially recommended by ACS in 2018 (17), the age to commence CRC screening was lowered from 50 years to 45 years for the entire at-risk population upon recommendation by USPSTF (18) (**Figure 1**). USPSTF recommendations generally trigger acceptance by CMS and other insurers to provide payment coverage. The reason for ACS and ultimately USPSTF recommendation was not data specific to NHB, but data showing overall increase in CRC under of 50 years for men and women in the population (12, 17, 18). The NHB CRC trends in CRC incidence under age 50 years has slightly increased from over 11 to over 12/100,000, whereas the NHW CRC incidence has increased from a low of 7 to 12/100,000, indicating that NHW are the larger driver for the recent population increase (17). AI/AN are also known to have among the highest rates for CRC incidence under 50 years (51). The one advantage of a broader recommendation of the 45 year commencement age is that it makes implementation easier *via* a more uniform message and policy for patients and providers.

Lowering CRC screening age to 45 years adds >19M average-risk persons to the screening pool (with 87M average-risk persons 50-74 years already in the screening pool) (52). With ~68% of 50-74 years and ~7% of 45-49 years obtaining screening previously, this policy change increases the unscreened population from ~27M to ~44M at-risk individuals, a 60% increase of pool size (52), and could constrain screening resources. The etiology of more CRCs under 50 years for both NHB and NHW is not known other than it is environmentally-driven; a targeted screening approach for under-age 50-years might be more ideal for resource efficiency if specific biomarkers are identified (13). Biomarker studies have been conducted nearly exclusively in NHW, with diagnostic accuracy of some existing biomarkers (e.g. mt-sDNA markers) not addressed with adequate power in other racial groups (3, 53). Furthermore, diagnostic accuracy has not been extended to those 45-49 years, with extrapolation of diagnostic accuracy from subjects aged ≥50 years. CRC screening utilization *via* colonoscopy is lower in younger age groups compared to older age groups (54), making high utilization among 45-49-year-old individuals challenging. All in all, the approach to screening for this enlarged population will take utilization of non-invasive and minimally-invasive strategies (see **Table 1**) to optimally screen the at-risk US population beginning at 45 years – rural and urban, all races and ethnicities, all socioeconomic strata, and insured and underinsured/non-insured (55).

## Discussion: Removing barriers and effective approaches for optimal CRC screening in racial groups

With modification to 45 years as initial CRC screening age, what test should be used to optimally screen 45-49 year-olds? At present, it is the best test that gets done (see **Table 1**) (53). Kaiser Permanente commenced screening in 45-49 year-old NHB individuals in 2018 *via* FIT after USMSTF recommended screening this racial group beginning at 45 years (11, 49). NHB aged 45-49 years, compared to NHB 50-54 years and NHW 50-54 years, had the lowest FIT completion rate despite FIT being mailed to 90% of eligible members. However, there were no automated electronic health reminders for this age group, and authors surmised that there was low provider awareness for screening in this age group and in NHB (49). This study reiterates that younger ages tend to have lower screening completion (54), and that there are provider, system, and patient barriers that may reduce CRC screening utilization (47). One study identified among non-screened average risk individuals that greatest barriers to screening with FIT/FOBT, multitarget-stool DNA (mt-sDNA) test, or colonoscopy were lack of knowledge, lack of provider recommendation, and suboptimal access (56). In those who had prior screening, barriers to completing the next FIT/FOBT or mt-sDNA test were lack of provider recommendation and lack of knowledge, and barriers to completing the next colonoscopy were psychosocial barriers and lack of provider recommendation (56, 57). NHB and Hispanic participants were more likely to report lack of knowledge and lack of provider recommendation than NHW individuals (56). Co-morbidities, which are more prevalent in NHB compared to NHW (42), adversely influence screening recommendations and completion (58). To facilitate screening among diverse populations with varying socioeconomic and social challenges, there needs to be contextually-informed, multi-level, multi-component interventions that target patients, providers, health systems, and communities (47, 55, 59). For instance, patients can benefit from navigation, providers can be educated to follow evidence-based guidelines, health systems can use electronic health records for systematic timely reminders to both providers and patients, and communities can address capacity, educational needs, and provide outreach (55). Policies need to be in place to remove barriers and promote uptake of evidence-based interventions, including removal of out-of-pocket costs for screening (55). Culturally-sensitive interactions between provider and patient may improve screening rates, including utilizing providers from the same race/ethnic background as the patient (60).

Navigation has proven to be a powerful tool to increase CRC screening rates in all populations (41, 61, 62). Not only does navigation increase screening utilization, it can erase disparity for CRC incidence and mortality between NHB and NHW, achieving health equity (41). There is a cost to navigation, which depends on the use of professional or volunteer navigators and the volume of patients for navigation. It is this author’s opinion that (a) navigation should be a component of CRC screening for those that need it, (b) should be cost-effective, (c) should be covered by insurance as part of CRC screening, (d) be principally used for colonoscopic screening completion, but components of navigation may be used for non-invasive screening completion, and (e) should be broadly available. Navigation is an ideal tool to increase CRC screening utilization and in particular for NHB and other disparate populations, and may be critical for screening rate improvement in the COVID-19 era (44).

Universal screening for CRC in at-risk individuals beginning at 45 years will require use of non-invasive and minimally-invasive tests. Along the screening continuum, non-invasive tests with abnormal results require follow-up colonoscopy; the two should be coupled together as the screening test (meaning any negative test is listed as completed, whether non-invasive or minimally invasive, but any abnormal non-invasive test is not completed until after the follow-up colonoscopy). Within Kaiser Permanente, 90% of patients with an abnormal FIT test were referred to colonoscopy, but only 52% completed a pre-procedure visit and 43% completed colonoscopy within 1 year (63). Clinic visit transition from primary care to gastroenterology may need to be optimized to prevent leakage of FIT-positive patients (63). Some insurers separate the follow-up colonoscopy after an abnormal non-invasive test and list it as “diagnostic” instead of “screening”, triggering co-pays from the patient and creating another (financial) barrier for screening completion. Professional organization advocacy has led to policy changes that at least partially rectified that issue.

Tools exist to remove barriers and increase screening utilization. Use of these tools will need to become the norm for broad implementation and success across all racial/ethnic groups.

## Data availability statement

The original contributions presented in the study are included in the article/supplementary material. Further inquiries can be directed to the corresponding author.

## Author contributions

JC conceived, wrote, and edited this manuscript. The author confirms being the sole contributor of this work and has approved it for publication.

## Funding

Supported by the United States Public Health Service (R01 CA258519) and the Rogel Cancer Center of the University of Michigan. The funders had no role in study design, data collection and analysis, decision to publish, or preparation of the manuscript.

## Conflict of interest

The authors declares that the research was conducted in the absence of any commercial or financial relationships that could be construed as a potential conflict of interest.

## Publisher’s note

All claims expressed in this article are solely those of the authors and do not necessarily represent those of their affiliated organizations, or those of the publisher, the editors and the reviewers. Any product that may be evaluated in this article, or claim that may be made by its manufacturer, is not guaranteed or endorsed by the publisher.

## Abbreviations

CRC, colorectal cancer; USPSTF, US Preventive Services Task Force; ACG, American College of Gastroenterology; ICSI, Institute for Clinical Systems Improvement; SSA, sessile serrated adenoma; NSAID, non-steroidal anti-inflammatory drug; ACS, American Cancer Society; USMSTF, US Multi-Society Task force of Colorectal Cancer; CT, computed tomography; ACP, American College of Physicians; FIT, fecal immunochemical test; FOBT, fecal occult blood test; NHB, non-Hispanic Black; NHW, non-Hispanic White; AI/AN, American Indian/Alaska Native; CMS, Centers for Medicaid and Medicare Services; MSI, microsatellite instability; mt-sDNA, multitarget-stool DNA test; COVID-19, coronavirus disease-2019.
